# Insights into the recognition of cyclic α-(1→6)-glucan by a solute-binding protein of an ABC transporter from *Tepidibacillus decaturensis*

**DOI:** 10.1016/j.jbc.2026.111346

**Published:** 2026-03-04

**Authors:** Shiho Takei, Wataru Saburi, Min Yao, Haruhide Mori, Toyoyuki Ose

**Affiliations:** 1 Faculty of Advanced Life Science, Hokkaido University, Sapporo, Japan; 2 Research Faculty of Agriculture, Hokkaido University, Sapporo, Japan

**Keywords:** ABC transporter, carbohydrate-binding protein, cycloisomaltooligosaccharide, isomaltooligosaccharide, isothermal titration calorimetry, sugar transport, X-ray crystallography

## Abstract

ABC transporters facilitate the translocation of various substrates across biological membranes. In prokaryotic ABC importers, the solute-binding protein, which selectively binds to a ligand, is incorporated into the functional complex. Cycloisomaltooligosaccharides (CIs) are produced from α-(1→6)-glucan by CI glucanotransferase and intracellularly degraded by CI-inducible dextranase. CIs are regarded as incorporated forms; however, their uptake mechanisms have not yet been elucidated. In this study, the solute-binding protein with a high affinity for CIs from *Tepidibacillus decaturensis* (TdCIBP) was discovered. TdCIBP showed the highest affinity for cycloisomaltoheptaose, followed by cycloisomaltooctaose and cycloisomaltononaose. TdCIBP also showed binding affinity for linear isomaltooligosaccharides (IGs) with a degree of polymerization ≥3 but preferred longer IGs. TdCIBP structures in complex with cycloisomaltooctaose and isomaltoheptaose were determined using X-ray crystallography at 1.6 Å and 1.9 Å resolutions, respectively. Of the modeled five d-glucosyl residues in isomaltoheptaose, the two d-glucosyl residues (the third and fourth residues from the reducing end) were bound to TdCIBP through numerous hydrogen bonding interactions in the same orientation as the corresponding d-glucosyl residues of cycloisomaltooctaose. The other d-glucosyl residues of isomaltoheptaose bind differently to the binding site than the corresponding d-glucosyl residues of cycloisomaltooctaose. As little difference was observed in the amino acid orientation of TdCIBP between the two complexes, cyclic and linear IGs were bound to TdCIBP by changing the combination of interacting amino acid residues. The high affinity to CIs and long IGs suggests that the ABC transporter cooperating with TdCIBP uptakes these sugars directly, contributing to sugar metabolism and minimizing ATP consumption.

## Introduction

ABC transporters are ubiquitous in diverse living organisms and facilitate the translocation of a wide spectrum of substrates across biological membranes. Constituting one of the most extensive protein superfamilies, ABC transporters are composed of two principal domains: a nucleotide-binding domain (NBD) and a transmembrane domain (TMD). However, for ABC importers found in prokaryotic systems, an additional domain, solute-binding protein (SBP), is incorporated into the functional complex. In gram-negative bacteria, SBPs are located in the periplasmic space, whereas in gram-positive bacteria and Archaea, they are either lipid anchored or covalently bound to the TMDs (1). SBP functions as a primary receptor that selectively binds to a ligand with high affinity, delivers it to the TMDs, and subsequently stimulates the ATPase activity of the NBDs (2). This ATP hydrolysis provides the energy required for the active transport of the ligand into the cytoplasm, concomitant with a large conformational change in NBDs. Therefore, SBPs are critical determinants of substrate specificity in the ABC transporter systems.

SBPs are characterized by a conserved domain architecture despite their low sequence similarity, and there is considerable diversity in their molecular mass (25–70 kDa) and in the number of functional units (monomers or dimers) (3). Structurally, SBPs are characterized by two N/C domains connected by a flexible hinge region, with a ligand-binding site located at the interdomain interface. In the unbound state, the domains exhibit conformational flexibility around the hinge, adopting an open conformation. Ligand binding induces a domain closure, thereby stabilizing the protein in a closed conformation, and this conformational transition is typically described as the “Venus flytrap” mechanism. SBPs recognize a wide range of target molecules, from simple ions to complex macromolecules; however, the molecular basis of their ligand recognition for certain substrates remains poorly understood.

Cycloisomaltooligosaccharides (CIs) are cyclic oligosaccharides composed of 7 to 17 α-(1→6)-d-glucosyl units (4). They exhibit high aqueous solubility and are known to inhibit the dextransucrase (Enzyme Commission [EC] 2.4.1.5)-catalyzed synthesis of α-(1→6)-glucan (dextran); as such, they are capable of impeding dental plaque formation (5). Decameric CI (CI10) forms an inclusion complex with Victoria Blue B (6). Enzymes related to the metabolism of CI have been reported; CIs are generally biosynthesized from dextran, which is produced from sucrose by dextransucrase, through an intramolecular transglycosylation catalyzed by cycloisomaltooligosaccharide glucanotransferase (CITase; EC 2.4.1.248) (7, 8, 9). Furthermore, α-(1→4)-glucan also serves as a starting material of CI production through a concerted reaction of 6-α-glucosyltransferase and CITase. These enzymes are extracellular enzymes in certain gram-positive bacterial strains, such as *Paenibacillus algidevorans* (*Niallia circulans* T-3040) and *Paenibacillus* sp. 598K. 6-α-Glucosyltransferase synthesizes isomaltooligosaccharyl moieties at the nonreducing ends of α-(1→4)-glucans *via* transglucosylation to provide substrates for CITase (10). CITase elongates the isomaltooligosaccharyl moiety, which is used as a substrate for CI production. *Paenibacillus* sp. 598K is capable of CI uptake and harbors an intracellular CI-degrading dextranase, which is inducible by CI supplementation, indicating that the strain metabolizes CI (11). *Thermoanaerobacter thermocopriae* possesses a multidomain enzyme comprising both CITase and 6-α-glucosyltransferase catalytic domains (12, 13, 14), suggesting the presence of a comparable metabolic pathway. Furthermore, within the genomic loci of *T. thermocopriae*, *Paenibacillus* sp. 598K and *P. algidevorans* putative SBP-encoding genes (GenBank accession: GBF74648.1 and National Center for Biotechnology Information [NCBI] reference sequence, WP_028992689.1 and GenBank accession: GBG10453.1, respectively) were found in proximity to the CITase gene. However, whether these putative SBPs bind cyclic or linear isomaltooligosaccharides (IGs) and how they recognize their substrates is unknown.

**Figure 1:**
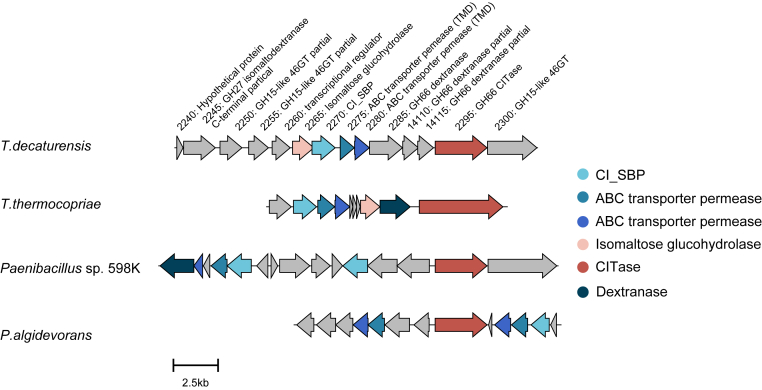
**Comparison of gene clusters of bacteria possessing CITase genes.** Gene organization in *Tepidibacillus decaturensis* and bacteria with CITase homologous proteins was compared using Clinker software (40). Homologous proteins sharing ≥30% amino acid sequence identity are depicted in the same color. CITase, cycloisomaltooligosaccharide glucanotransferase.

The rod-shaped gram-negative bacterium, *Tepidibacillus decaturensis* (15), also encodes a putative SBP gene (TdCIBP; locus tag, U473_RS02270; NCBI reference sequence, WP_201024125.1) located upstream of the putative CITase gene (locus tag, U473_RS02295; NCBI reference sequence, WP_068722958.1) (Fig. 1). Notably, recent biochemical studies have demonstrated that *T. decaturensis* possesses an α-glucan 4(6)-α-glucosyltransferase (46GT) that converts α-(1→4)-glucans into α-(1→6)-glucans, indicating that this organism is capable of endogenously generating α-(1→6)-linked glucans (16). These findings suggest that *T. decaturensis* harbors a metabolic framework for the synthesis and utilization of α-(1→6)-glucan–derived carbohydrates, potentially including CIs. The amino acid sequence of TdCIBP shares 37% and 58% identity with the putative SBPs from *Paenibacillus* sp. 598K and *T. thermocopria*, respectively. Despite this circumstantial evidence, it remains unclear how *T. decaturensis* selectively recognizes and imports α-(1→6)-glucan–derived carbohydrates. Addressing this question is essential for understanding how molecular recognition strategies underpin its carbohydrate utilization biology. We hypothesized that these SBPs are capable of recognizing CIs and functioning as a CI-binding protein (CIBP). In the present study, we aimed to elucidate the ligand specificity and structural basis of substrate recognition by TdCIBP. Therefore, in the present study, we prepared recombinant TdCIBP and conducted biochemical and structural characterizations to experimentally identify CIBP. Isothermal titration calorimetry (ITC) demonstrated that TdCIBP binds exothermically to CI with the oligomerization numbers of 7 to 9 (CI7−CI9) and linear IGs with the oligomerization numbers of 4 to 7 (IG4–IG7). The strongest binding was observed with CI7, and there was almost no change in entropy. Binding to CI8 and CI9 was also stronger than binding to IGs and was characterized by entropy-favorable profiles, whereas binding to IGs showed entropy-unfavorable profiles. Crystal structure analyses of TdCIBP in complex with CI8 or IG7 indicated extensive interactions for recognizing the fixed conformations of each ligand. These properties clearly explain the preference of TdCIBP for CIs and IGs composed of a higher number of glucose units, which can be relevant to its nutrient-obtaining strategy.

## Results

### Preparation of recombinant TdCIBP and its oligomeric state analysis

We established a protocol for expressing and purifying recombinant TdCIBP. The construct used for all experiments comprised residues 25 to 432, excluding the N-terminal signal peptide predicted by SignalP (https://services.healthtech.dtu.dk/services/SignalP-6.0/). At the C terminus, 6× His was added for purification. Recombinant TdCIBP was purified by nickel ion affinity chromatography, followed by size-exclusion chromatography (SEC). To assess the oligomeric state, an SEC-coupled multiangle light scattering (MALS) experiment was conducted (Fig. S1), and the SEC profile indicated two prominent peaks. At the first eluted peak position (9.2 ml), the molecular mass was calculated as 99 ± 1.4 kDa, consistent with the theoretical mass of a dimer (92 kDa). The second peak (10.2 ml) was larger than the first, and the molecular mass was calculated as 46 ± 0.3 kDa, which matched the theoretical mass of a monomer (46 kDa). These results indicated that under the test conditions, recombinant TdCIBP exists predominantly as a monomer, with a minor population forming dimers.

### Binding specificity of TdCIBP toward α-(1→6)-linked glucooligosaccharide ligands

Ligand-binding affinity and specificity of TdCIBP were investigated with ITC using CI7−CI9, a series of IGs (IG*n*; *n*, degree of polymerization, *n* = 2–7), d-glucose, and γ-cyclodextrin as ligands (Table 1). Of the ligands tested, CI9, CI8, CI7, IG7, IG6, IG5, and IG4 exhibited clear exothermic binding profiles (Fig. S2). The dissociation constant (*K*_*D*_), calculated using the one-set-of-sites model, demonstrated that the binding of CIs was stronger than that of linear ones. For CIs, the *K*_*D*_ values ranged from 0.045 to 0.11 μm, with CI7 exhibiting the highest affinity (*K*_*D*_ = 0.045 ± 0.002 μm), followed by comparable constants with CI8 (0.11 ± 0.02 μm) and CI9 (0.11 ± 0.02 μm). The *K*_*D*_ value of TdCIBP was lower for longer IGs in IG4–IG7 (ranging from 0.32 to 1 μm). Although TdCIBP also exhibited a measurable exothermic reaction upon binding to IG3, it was not possible to accurately fit the thermodynamic data to the one-set-of-sites model (Fig. S2). No marked heat generation was detected for IG2, d-glucose, or γ-cyclodextrin. The results indicated that effective binding requires IGs with a degree of polymerization ≥3 to establish sufficient molecular contacts. To our knowledge, no SBP with specificity for α-(1→6)-linked glucans has been reported previously, and this result showed that TdCIBP is a unique member within this protein family.

**Table 1:** Affinity and thermodynamic parameters for ligand binding to TdCIBP at 25 °C as determined using ITC

Ligand	*K*_*D*_ (μm)	Stoichiometry	Δ*G* (kJ/mol)	Δ*H* (kJ/mol)	-*T*Δ*S* (kJ/mol)
CI9	0.11 ± 0.02	0.65 ± 0.037	−40 ± 0.1	−34 ± 0.3	−6 ± 0.2
CI8	0.11 ± 0.02	0.76 ± 0.002	−40 ± 0.5	−28 ± 0.8	−12 ± 1
CI7	0.045 ± 0.002	0.79 ± 0.004	−42 ± 0.1	−43 ± 0.1	1 ± 0.1
IG7	0.32 ± 0.06	0.78 ± 0.02	−37 ± 0.5	−47 ± 1.5	10 ± 2
IG6	0.68 ± 0.02	0.9 ± 0.000001	−35 ± 0.4	−46 ± 0.9	11 ± 1
IG5	1.1 ± 0.01	0.9 ± 0.009	−34 ± 0.4	−45 ± 1.5	10 ± 2
IG4	0.93 ± 0.008	0.91 ± 0.007	−35 ± 1	−44 ± 2.0	9 ± 3
IG3^a^	(1.8 ± 0.006)	(1.3 ± 0.006)	(−27 ± 0.3)	(−33 ± 1.7)	(6 ± 2)
IG2	No heat generation	No heat generation	No heat generation	No heat generation	No heat generation
d-glucose	No heat generation	No heat generation	No heat generation	No heat generation	No heat generation
γ-CD	No heat generation	No heat generation	No heat generation	No heat generation	No heat generation

Values represent mean ± SD from three independent ITC experiments.

a Accurate thermodynamic values are difficult to determine because the presence of heterogeneous binding modes obscures reliable estimation of the stoichiometry.

### Crystal structure analysis of TdCIBP in complex with CI8 or IG7

**Figure 2:**
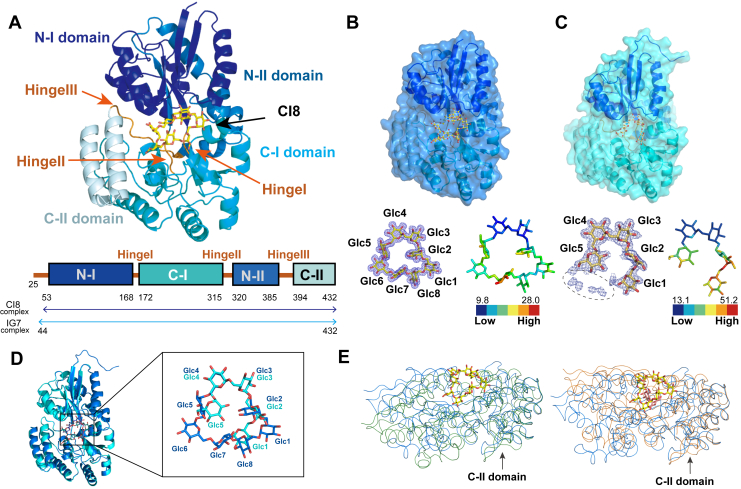
**Overall structures of TdCIBP and comparison with related proteins.***A*, overall structure of the TdCIBP–CI8 complex. The *upper panel* shows the crystal structure of the TdCIBP–CI8 complex in *ribbon representation*. The N-domain, C-domain, and hinge region of TdCIBP are colored *blue*, *cyan*, and *orange*, respectively. The bound CI8 molecule is shown as *yellow sticks*. The *lower panel* depicts the domain organization of TdCIBP, with colors corresponding to those in the *upper panel*. The regions modeled in the crystal structures of TdCIBP–CI8 and TdCIBP–IG7 are indicated with *arrows*. *B* and *C*, complex structures of TdCIBP bound to CI8 (*B*) and IG7 (*C*). The *upper panels* show *ribbon and surface representations* of each ligand. The *lower left panels* display the composite omit (*F*o–*F*c) electron density maps for CI8 (*B*) and IG7 (*C*) bound to TdCIBP, contoured at 2.5σ for CI8 and 1.5σ for IG7. In the IG7 complex, additional electron density suggestive of d-glucose units was observed adjacent to both ends of the modeled ligand and is indicated by *dashed outlines*; however, this density was insufficient for reliable model building and therefore not included in the final model. The *lower right panels* show the corresponding *B*-factor distributions of the bound ligands CI8 and IG7. *D*, structural comparison of ligand-bound complexes. Superimposition of TdCIBP bound to CI8 (*blue*) and IG7 (*cyan*) is shown in *ribbon representation* (*left*). The *right panel* provides an enlarged view highlighting the bound isomaltooligosaccharides. *E*, comparison of TdCIBP with tmMBP3; the *left panel* shows TdCIBP (*blue*) superimposed on the unliganded tmMBP3 structure (*green*; PDB ID: 6DTR), whereas the *right panel* shows TdCIBP compared with tmMBP3 bound to maltose (*orange*; PDB ID: 6DTQ). The structural alignment was performed based on the C-II domain. PDB, protein data bank; TdCIBP, cycloisomaltooligosaccharide-binding protein from *Tepidibacillus decaturensis*.

Crystallization trials demonstrated that TdCIBP did not crystallize in the absence of a ligand, which is consistent with the previously suggested conformational flexibility between the domains (17, 18). In contrast, well-diffracted crystals were obtained in the presence of CI8 or IG7, enabling structural analyses. We determined the crystal structures of TdCIBP in complex with CI8 at 1.6 Å and IG7 at 1.9 Å resolutions (Table 2). In each crystal form, an asymmetric unit contained a single protomer. The electron density corresponding to the N-terminal region (residues 25–52 in the CI8 complex; residues 25–43 in the IG7 complex) was not visible; the other region (residues 53–432 or 44–432) was clear and thus successfully modeled (Fig. 2, *A–C*). In both complexes, composite omit map calculations indicated connected density in the interdomain cleft, allowing the modeling of all eight d-glucose units for CI8 and five units for IG7 (Fig. 2, *A–C*). The d-glucose residues in IG7 were numbered sequentially from the reducing end to the nonreducing end. For consistency, CI8 was numbered according to the orientation of glucose units in IG7. The overall architectures of the CI8- and IG7-bound TdCIBP structures were nearly identical, with an RMSD of 0.237 Å (Fig. 2*D*). These observations established that TdCIBP recognizes cyclic/linear IGs in the canonical interdomain pocket without major conformational changes.

**Table 2:** Data collection and refinement statistics

Data collection	TdCIBP/CI8	TdCIBP/IG7
Space group	*P*2_1_2_1_2_1_	*P*2_1_2_1_2_1_
Cell dimensions (Å)	69.33, 78.94, 83.40	62.29, 68.70, 108.41
Wavelength (Å)	1.00	1.00
Resolution (Å)^a^	50.0–1.60 (1.70–1.60)	46.2–1.90 (2.02–1.90)
*R*_meas_	0.206	0.234
*I*/σ*I*	21.9 (1.98)	8.87 (1.63)
Wilson *B*-factor	17.18	25.3
Completeness (%)	100	100
Redundancy	22.9	10.7
Refinement		
Resolution (Å)^a^	44.18–1.60 (1.63–1.60)	46.15–1.90 (1.95–1.90)
No. of reflections	60,951 (2,836)	37,369 (1,130)
*R*_work_/*R*_free_	0.169/0.196 (0.244/0.264)	0.167/0.200 (0.298/0.334)
No. of atoms		
Protein	2,925	3,002
Ligand	88	56
Water	412	370
*B*-factor	20.01	24.66
Protein	18.64	23.29
Ligand	15.37	27.71
Water	30.74	35.34
RMSD		
Bond lengths (Å)	0.010	0.011
Bond angles (°)	1.0	1.1
Ramachandran plot		
Favored (%)	98.9	99.2
Outliers (%)	0.3	0

a Highest resolution shell is shown in parenthesis.

### Comparison with the known structures of CI8

To compare the CI8 conformation observed in the TdCIBP-bound structure with previously reported CI structures, we examined the available atomic structure of CI8, which was determined in complex with CITase (Protein Data Bank [PDB] ID: 3WNO (19)). In this structure, three CI8 molecules are bound to the enzyme. Superposition of these three CI8 molecules with the CI8 bound to TdCIBP indicated that the overall cyclic topology is well conserved across all structures (Fig. S3*A*). In contrast, local differences were observed in the orientations of individual d-glucose residues, whereas the global ring architecture remained similar.

Previously, small-angle X-ray scattering (SAXS) analyses of CI8 demonstrated that free CI8 exists as an ensemble of multiple compact conformations rather than a single rigid structure or extended structures (20). The Kratky plot was calculated from the atomic coordinate of TdCIBP-bound CI8, showing a maximum at a similar *q* value, indicating a comparable degree of compactness (Fig. S3*B*). Moreover, the radius of gyration (*R*g) calculated from the TdCIBP-bound CI8 structure (*R*g = 6.9 Å) closely matched the *R*g reported for free CI8 in solution (*R*g = 6.9 Å) (20).

### Overall structures of TdCIBP in complex with CI8 or IG7

The overall architecture of TdCIBP adopts the canonical SBP fold, comprising two globular N/C domains connected by hinge regions (I–III) (Fig. 2*A*). The N-terminal domain includes residues 53 to 168 (N-I domain) and 320 to 385 (N-II domain), whereas the C-terminal domain comprises residues 172 to 315 (C-I domain) and 394 to 432 (C-II domain). These domains are connected *via* three hinge segments: hinge I (residues 169–171), hinge II (residues 316–319), and hinge III (residues 386–393). The N-terminal domain features a five-stranded β-sheet flanked by nine α-helices, and the C-terminal domain contains a three-stranded β-sheet similarly flanked by nine α-helices. Based on these features, TdCIBP was classified within the structural cluster D–I of SBPs (17). A structure similarity search using Foldseek (21) indicated that TdCIBP shares high structural homology with other SBPs known to bind carbohydrate ligands, including α- and β-glucosides (Table 3).

**Table 3:** Results of the structural similarity search

PDB ID (chain ID)	*E* value	Sequence identity	Name	Organism	Binding specificity	Ref.
3JZJ (A)	7.53E-32	23.3	GacH	*Streptomyces glaucescens*	α-acarbose	(43)
8ART (B)	2.93E-33	26	MalE	*Streptomyces scabiei*	Maltose	TBP
8ART (A)	1.40E-32	25.8	MalE	*Streptomyces scabiei*	Maltose	TBP
5CI5 (B)	6.96E-31	23.7	Tlet_1705	*Pseudothermotoga lettingae TMO*	α-D-Tagatose	TBP
4QSE (B)	1.08E-31	27.9	ATU4361	*Agrobacterium fabrum str. C58*	Glycerol	TBP
5YSE (A)	1.40E-29	21.1	SO-BP	*Listeria innocua Clip11262*	β-1,2-Glucooligosaccharide	(44)
6DTR	1.17E-29	25.2	tmMBP3	*Thermotoga maritima MSB8*	Apo, maltotetraose	(18)
7BVT (A)	3.67E-29	23.9	CMMBP	*Arthrobacter globiformis*	Cyclobis-(1→6)-α-maltosyl	(22)
3UOR (B)	5.17E-28	21.2	Xac-MalE	*Xanthomonas citri pv. citri str. 306*	Apo	(45)
1EU8 (A)	1.69E-26	23.2	TMBP	*Thermococcus litoralis*	Trehalose/maltose	(46)

TBP, to be published.

Proteins mentioned in the main text are underlined.

To further analyze the conformational state of TdCIBP, we compared TdCIBP with maltose-binding protein from *Thermotoga maritima* (tmMBP3), a well-characterized SBP with both apo- (open) and ligand-bound (closed) structures (18). Structure superimposition of TdCIBP onto tmMBP3, based on Cα atoms of the C-terminal 40 residues, demonstrated that the TdCIBP structure is similar to the open conformation of tmMBP3 as the apo form (Fig. 2*E*). Intriguingly, similar open conformations have been observed in other SBPs in complex with cyclic oligosaccharides, including cyclobis-(1→6)-α-maltosyl (CMM), a cyclic glucotetrasaccharide, with alternating α-(1→4)- and α-(1→6)-linkages and β-cyclodextrin (22, 23). These observations suggest that SBPs recognizing cyclic or bulky carbohydrate ligands may undergo limited domain closure upon ligand binding, in contrast to the pronounced hinge-bending motions observed in SBPs that bind to linear oligosaccharides.

### Location of the ligand-binding site

A composite omit map (*Fo–Fc*) of the two crystal forms unambiguously indicated the connected electron density corresponding to each ligand, CI8 or IG7. For CI8, each oligosaccharide adopts a compact folding, with each chair-form pyranose ring connected *via* α-(1→6)-linkages in staggered geometries. The well-ordered electron density allowed precise modeling of each d-glucose unit (Fig. 2*B*). For IG7, the electron density of IGs was also observed in the crystal of TdCIBP with IG7. However, because of the fragmentation of the density, only five of the seven d-glucose units could be modeled (Fig. 2*C*). The curvature of recognized IG7 is reminiscent of that of CI8. Additional d-glucose units were likely present adjacent to both ends (Glc1 or Glc5), but the electron density in these regions was insufficient for modeling (Fig. 2*C*). The ligand-binding cleft is formed by these three regions: the N-terminal domain (N-I and II domains), the C-terminal domain (C-I domain), and the intervening hinge regions (hinge III). The ligand-binding cleft comprises three structural regions that contribute to distinct interactions with CI8 and IG7 (summarized in Table S1). Polar residues from the N-domain form direct or water-mediated hydrogen bonds, nonpolar residues from the C-domain are involved in hydrophobic stacking interactions mediated by the three Trp residues (Trp217, Trp275, and Trp290), and the hinge region provides additional polar contact. Fourteen amino acid residues of TdCIBP were involved in ligand interactions in both CI8 and IG7 (summarized in Table S1).

### CI8 recognition mode

**Figure 3:**
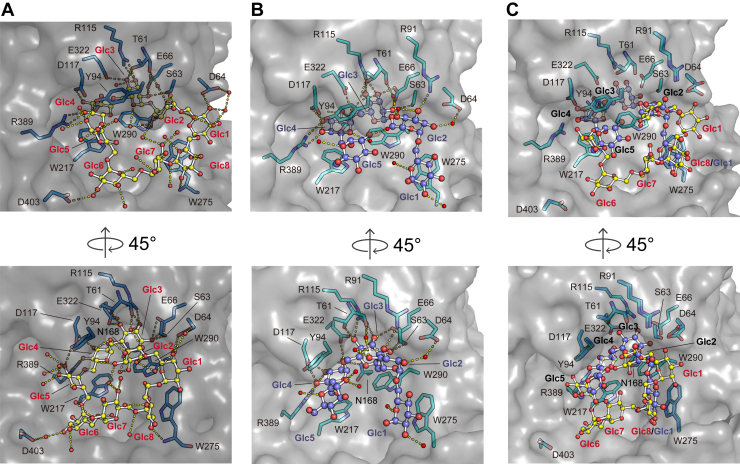
**Binding modes of TdCIBP with CI8 and IG7.** Detailed views of the interactions between TdCIBP and CI8 (*A*) or IG7 (*B*) at the ligand-binding sites. The interacting residues of TdCIBP are shown as *sticks*, each ligand is represented by *a ball and a stick*, and *dashed lines* indicate potential hydrogen-bonding contacts within a distance of 3.3 Å. The *right panels* show the same binding sites rotated by 45° relative to those in the *left panels*. *C*, superimposed representation of the side chains from both TdCIBP residues and each ligand (*ball-and-stick model*). CI8 is shown with oxygen (*red*) and carbon (*yellow*), whereas IG7 is shown with oxygen (*red*) and carbon (*purple*). TdCIBP, cycloisomaltooligosaccharide-binding protein from *Tepidibacillus decaturensis.*

Hydrogen bonds and hydrophobic interactions are distributed throughout the eight d-glucose units (Glc1–Glc8) (Fig. 3*A*). Glc1 forms a hydrogen bond with Asp64 and a nonpolar interaction with Trp275, and Glc2 establishes a water-mediated hydrogen bond with Thr61. Glc3 is extensively stabilized through direct hydrogen bonds with six residues (Glu66, Ser63, Arg115, Glu322, Thr61, and Arg115), a water-mediated hydrogen bond with Glu322, and a nonpolar interaction with Trp290. Glc4 engages in hydrogen bonds with Asn168, Asp117, and Arg389, as well as a nonpolar interaction with Trp217. Glc5 forms a hydrogen bond with Tyr94 and a hydrophobic interaction with Trp217, whereas Glc6 forms a water-mediated hydrogen bond with Asp403. No specific interactions were observed for Glc7. Glc8 forms both a hydrogen bond and a nonpolar interaction with Trp275. Seven ordered water molecules were observed within CI8, mediating nine intraligand hydrogen bonds among the d-glucose units (Fig. S4). The *B*-factor distribution of the CI8 atoms (Fig. 2*B*) reflected the number of interactions each residue forms: Glc1, Glc3, Glc4, Glc6, and Glc8 showed lower *B*-factors, corresponding to extensive contacts and reduced mobility, whereas Glc2, Glc5, and Glc7 exhibited relatively higher *B*-factors, consistent with fewer direct interactions and greater flexibility.

### IG7 recognition mode

The recognition mechanism of IG7, using 14 amino acid residues, is similar to that of CI8 recognition (Fig. 3*B*). Glc1 forms a nonpolar interaction with Trp275. Glc2 establishes one direct hydrogen bond with Arg91 and two water-mediated hydrogen bonds with Asp64 and Thr61. Glc3 forms five direct hydrogen bonds (Glu66, Ser63, Arg115, Thr61, and Glu322), two water-mediated hydrogen bonds (Asn168 and Glu322), and a nonpolar interaction with Trp290. Glc4 forms hydrogen bonds with Asn168, Glu322, Asp117, and Arg389 and nonpolar interactions with Trp290 and Trp217. Glc5 forms a water-mediated hydrogen bond and a nonpolar interaction with Tyr94. The *B*-factor distribution of the IG7 atoms is similarly correlated with the number of contacts; Glc3 and Glc4 display lower *B*-factors because of extensive interactions, whereas Glc1, Glc2, and Glc5 exhibit higher *B*-factors, reflecting fewer direct contacts and higher flexibility (Fig. 2*C*). Seven water molecules were also identified within IG7, mediating four intraligand hydrogen bonds (Fig. S3*B*).

### Comparison of the two ligand-binding modes

A structural comparison between the CI8- and IG7-bound complexes indicated that, of the 14 residues of TdCIBP involved in ligand interactions, only Asp64 exhibited a change in side-chain orientation, whereas all other interacting residues were well superimposed (Fig. 3*C*). Comparison of the bound ligands showed that the d-glucose units located in the inner part of the binding cleft (Glc3 and Glc4) adopt nearly identical orientations. In both complexes, Glc3 and Glc4 commonly form multiple direct hydrogen bonds with the side chains of surrounding residues. These interactions suggest that this region is tightly recognized by TdCIBP and contributes to fixing the locations of the oligosaccharides. These two d-glucose residues consistently exhibit low *B*-factors (Fig. 2, *B* and *C*), indicating they being tightly recognized.

In contrast, Glc1, Glc2, and Glc5 showed conformational differences (Figs. 2*D* and 3*C*), which result in changes in the sets of interacting residues involved in contact formation. These d-glucose units displayed pronounced differences in orientation, accompanied by changes in their interacting residues. For Glc8 of CI8 and Glc1 of IG7, the hydrophobic stacking of the pyranose ring with Trp275 is conserved in both complexes, whereas an additional hydrogen bond with Asp64 is observed only in CI8. For Glc2, both complexes form a water-mediated hydrogen bond with Thr61; however, Glc2 of IG7 forms an additional direct hydrogen bond with Arg91 and a water-mediated hydrogen bond with Asp64. For Glc5, CI8 forms a hydrophobic interaction with Trp217 and a hydrogen bond with Tyr94, whereas for IG7, Glc5 exhibits only a hydrophobic interaction with Tyr94. Therefore, the difference in the orientations of the d-glucosyl residues between CI8 and IG7 (Fig. 2*D*) can be attributed not only to the intrinsic structural properties of cyclic and linear IGs but also to the small conformational change of the side chain of Asp64 (Fig. 3*C*).

IG7 forms more water-mediated indirect interactions than CI8 (summarized in Table S1). Furthermore, the d-glucosyl residues that are not well superimposed between the two structures (Glc1, Glc2, and Glc5) likely retain higher conformational flexibility in IG7. Notably, as an α-(1→6) linkage has a higher degree of rotational freedom than the α-(1→4) linkage (20), these regions in IG7 can be adopted as low-energy torsional conformations to be recognized by TdCIBP.

## Discussion

SBPs are key determinants of substrate specificity in ABC transporters; however, only crystal structures with a limited number of SBPs in complex with ligands have been reported (3, 17). SBPs are classified into clusters A–G (17, 24). Among them, sugar-binding SBPs fall into clusters B, D, and G, which typically recognize monosaccharides, oligosaccharides, and polysaccharides (alginates), respectively. Members of cluster D have been shown to recognize α-(1→4)-linked glucooligosaccharides, such as maltose and cyclodextrin, which serve as representative substrates. Despite growing interest in the metabolic pathways of α-(1→6)-linked oligosaccharides such as IGs, driven by their industrial relevance, no structural study has yet described an SBP in complex with an IG.

We focused on genes annotated as SBPs located in proximity to CITase genes and investigated the possibility of discovering a novel SBP that recognizes CIs. A putative SBP gene from *T. decaturensis* was used, and the corresponding recombinant protein, which was designated as TdCIBP, was characterized. Structural analysis was conducted using crystallography, and ligand-binding studies were conducted using calorimetry to elucidate the molecular features and substrate-recognition mechanism. Of the ligands tested, CI7 exhibited the highest binding affinity, followed by CI9≃CI8 and IG7 (Table 1). Notably, CI8 and CI9 displayed an entropy-favorable binding profile, whereas IGs exhibited entropy-unfavorable profiles, and the balance of entropy for CI7 binding was approximately zero. This thermodynamic signature reflects ligand conformational properties, as flexible linear oligosaccharides lose conformational entropy upon binding (23). In contrast, the cyclic architecture of CI likely incurs a smaller entropy penalty because of its inherently rigid and preorganized structure, resulting in a net favorable entropy contribution. Previous structural analyses of CIs have demonstrated that they adopt more compact and relatively inflexible ring conformations in solution than their linear counterparts (20). The well-defined electron density observed for CI8 by structural analysis indicates a stable bound conformation. The conformation of CI8 observed in the TdCIBP-bound crystal structure can also be evaluated in the context of solution studies of free CI8. Albeit the conformational heterogeneity, the Kratky plot of free CI8 previously reported exhibits a well-defined maximum, indicating that CI8 maintains a compact structure in solution rather than behaving as a random coil (Fig. S4) (20). Consistent with this observation, the Kratky plots calculated from the atomic coordinates of TdCIBP-bound CI8 correspond to one of the compact conformations accessible to CI8 in solution, rather than a highly distorted or artificially constrained state.

In contrast, IG7 binding is associated with a markedly more favorable enthalpic contribution than CI binding, as indicated by the large negative Δ*H*. IG7 forms a greater number of water-mediated indirect interactions with TdCIBP than CI8 (Table S1). These hydration-mediated interactions likely provide enthalpic stabilization that compensates for the unfavorable entropy change accompanying IG binding.

In comparison, the binding positions of CI7 and CI9 are unclear because their complex structures were not examined in this study. However, the affinity of CI9 was comparable to that of CI8, suggesting that the additional d-glucose unit does not substantially affect the interactions within the major binding region (Glc2–Glc5). CI7 exhibited the highest binding affinity of the three CIs, raising the possibility that it adopts an even more favorable binding mode, although this awaits further structural analysis for confirmation.

The electron density and *B*-factor values of five of the seven d-glucose units of IG7 provided insights into the binding hierarchy across the major and minor binding subsites in TdCIBP. The gradual decrease in affinity from IG7 (*K*_*D*_ = 0.32 μm) to IG5 (*K*_*D*_ = 1.1 μm) can be rationalized by the presence of residual density in the composite omit map, corresponding to two additional d-glucosyl residues and suggesting that all seven units contribute to binding (Fig. 2*C*). Based on the refined atomic *B*-factors (Fig. 2*C*), the relative binding priorities of the modeled d-glucosyl residues were ranked as Glc4 = Glc3 > Glc5 > Glc2 > Glc1 (Fig. 2*C*). The highest *B*-factor observed for Glc1 indicates that this terminal residue contributes minimally to the interaction out of the five units, which is consistent with the comparable affinities of IG5 (*K*_*D*_ = 1.1 μm) and IG4 (*K*_*D*_ = 0.93 μm).

Notably, the thermograms of IG4 and IG3 exhibited clear differences in the ITC experiments (Fig. S2). The binding of IG3 could not be reliably fitted to a one-set-of-sites model, likely because multiple IG3 molecules can occupy distinct subsites within the 8-d-glucose binding groove of a single TdCIBP molecule. Consequently, reliable stoichiometric parameters could not be derived from the IG3 titration, although the measurable heat release confirmed that binding had occurred. In contrast, titration experiments using IG2 or d-glucose did not yield detectable heat changes, indicating that three d-glucosyl units are required for detectable binding. Structural analyses indicated that the Glc2–Glc5 region constitutes the major interaction sites in TdCIBP, suggesting that four contiguous d-glucosyl residues are necessary for stable 1:1 complex formation. This interpretation is consistent with the comparable affinities observed for IG4 and longer IGs and provides a structural basis for understanding the substrate length requirement of TdCIBP (Table 1). The residues forming these subsites are highly conserved among related SBPs, as described below.

**Figure 4:**
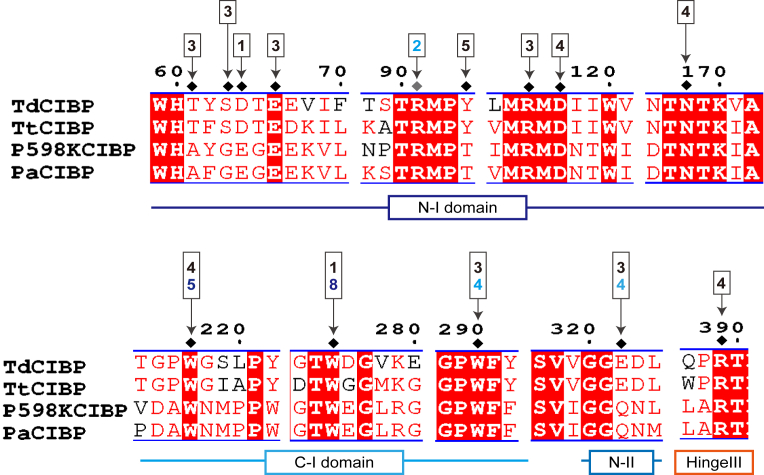
**Multiple sequence alignment of CIBPs.** The alignment was generated using MAFFT (41, 42). *Black diamonds* indicate residues involved in the recognition of CI8 and IG7, whereas the *gray diamonds* indicate Arg91, which specifically contributes to IG7 recognition. The number of d-glucose units involved in the interactions with both CI8 and IG7 are shown in *black*; those interacting with CI8 only are shown in *blue*, and those interacting with IG7 only are shown in *cyan*. Amino acid positions corresponding to TdCIBP are shown above the alignment. TtCIBP, P598KCIBP, and PaCIBP, derived from *Thermoanaerobacter thermocopriae*, *Paenibacillus* sp. 598K, and *Paenibacillus algidevorans*, respectively, are solute-binding proteins encoded within the same operon as CITase. CIBP, CI-binding protein; CITase, cycloisomaltooligosaccharide glucanotransferase; TdCIBP, cycloisomaltooligosaccharide-binding protein from *Tepidibacillus decaturensis*.

To evaluate the conservation of residues critical for substrate recognition, we compared the amino acid sequences of putative SBPs from several bacteria possessing the CITase gene, including *T. thermocopriae*, *Paenibacillus* sp. 598K, and *P*. *algidevorans*, with that of TdCIBP (Fig. 4). In particular, several aromatic and polar residues, Glu66 (recognizing Glc3), Arg91 (Glc2), Arg115 (Glc3), Asp117 (Glc4), Asn168 (Glc4), Trp217 (Glc4), Trp275 (Glc1), Trp290 (Glc3), and Arg389 (Glc4), are conserved across these species. These residues mediate key stacking and hydrogen-bonding interactions in TdCIBP, and their strong conservation among homologs indicates that the Glc2–Glc5 binding region identified in the crystal structures is a shared and essential recognition site. All interacting residues are strictly conserved, except for the Glc5 subsite, where Tyr94 in TdCIBP is replaced by Thr in some homologs, a chemically similar substitution that likely conserves hydrogen-bonding capability. This conservation supports the conclusion that the Glc2–Glc5 region is essential for substrate recognition by TdCIBP and related SBPs.

**Figure 5:**
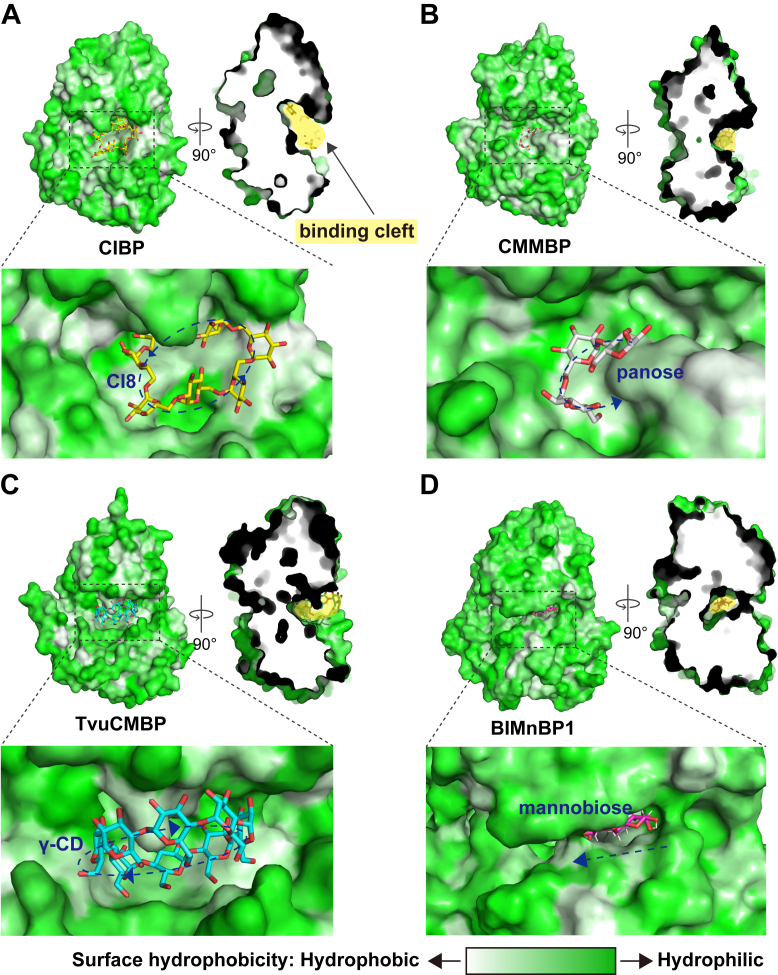
**Comparison of substrate-binding clefts among SBPs.** Surface representations of TdCIBP (*A*), CMMBP (*B*; PDB ID: 7BVT), TvuCMBP (*C*; PDB ID: 2ZYM), and BIMnBP1 (*D*; PDB ID: 6I5W) are shown. The molecular surfaces are colored according to hydrophobicity. The *blue dashed arrows* indicate the orientation of the ligand from the nonreducing end to the reducing end. CI8 and γ-cyclodextrin are cyclic ligands; therefore, they lack defined reducing and nonreducing ends. For a comparison with the linear oligosaccharides, the orientation of the cyclic ligands was assigned by designating one d-glucose residue as a pseudo-reducing end, defining it sequentially toward the pseudo-nonreducing end. PDB, protein data bank; SBP, solute-binding protein; TdCIBP, cycloisomaltooligosaccharide-binding protein from *Tepidibacillus decaturensis*; TvuCMBP, *Thermosipho vulcanus* cyclic maltosyl-binding protein.

We compared the architectures of the substrate-binding clefts of several SBPs, including TdCIBP, CMMBP (22), *Thermosipho vulcanus* cyclic maltosyl-binding protein (TvuCMBP), and BlMnBP1 (25) (Fig. 5). Of these, CMMBP, which binds to cyclic oligosaccharides, showed high structural similarity to TdCIBP. Although TvuCMBP recognizes γ-cyclodextrin, it exhibits low overall similarity to TdCIBP. In contrast, BlMnBP1 interacts with linear oligosaccharides. TdCIBP accommodates CI8 in a flattened, extended conformation within a shallow and broad binding cleft. The ligand binds parallel to the long axis of the TdCIBP molecule. A similar arrangement was observed in CMMBP, where CMM is bound in an extended manner within a comparably shallow cleft, also oriented parallel to the major axis of the protein. In contrast, TvuCMBP captures γ-cyclodextrin in a perpendicular orientation relative to its long axis. Its binding cleft is narrower and as shallow as those of TdCIBP and CMMBP, and the ligand fits into the cleft in a plug-like fashion rather than lying across the surface. SBPs that bind to linear oligosaccharides, such as BlMnBP1, exhibit a markedly different architecture: a long, narrow cleft that runs horizontally between the N- and C-lobes of the protein. This deep, tunnel-like cavity tightly accommodates the elongated sugar chains. Mannobiose is bound perpendicularly to the major axis of the protein and occupies this enclosed channel.

**Figure 6:**
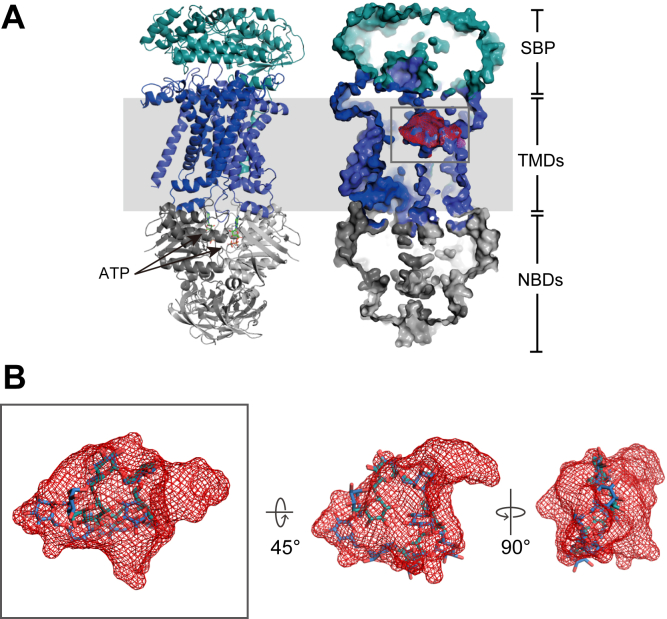
**Predicted structure of TdCIBP–ABC transporter complex.** The complex comprising the solute-binding protein (SBP), transmembrane domains (TMDs), nucleotide-binding domains (NBDs), and ATP was predicted using the ProteinX server (36). The predicted model exhibited confidence scores of pTM = 0.88 and ipTM = 0.87. *A*, overall structure of the TdCIBP–ABC transporter complex shown in *ribbon representation* (*left*) and *surface representation* (*right*). The SBP, TMDs, and NBDs are colored in *cyan*, *blue*, and *gray*, respectively. The two ATP molecules bound to the NBDs are depicted as *green sticks* in the *left* model. The substrate-binding pocket identified by POCASA is highlighted by a *mesh* in the TMD region (*boxed area*). *B*, enlarged views of the substrate-binding pocket shown in (*A*). CI8 and IG7, derived from the crystal structures of TdCIBP in complex with CI8 or IG7, are superimposed into the predicted pocket and shown from three orientations (original view, 45° rotation, and 90° rotation) to illustrate the spatial relationship between the ligands and the pocket. ipTM, interface pTM; pTM, predicted template modeling; TdCIBP, cycloisomaltooligosaccharide-binding protein from *Tepidibacillus decaturensis*.

We next explored the relationship between substrate size and downstream transport by comparing the substrate-binding cavity dimensions of ABC importers. Although the substrate specificity in these systems is largely defined by the cognate SBP, the size of the substrate is further constrained by the dimensions of the substrate-binding site within the transporter (26). Genes encoding carbohydrate-metabolizing enzymes and transporter TMDs were identified in the genomic region surrounding TdCIBP (Fig. 1). In contrast, NBDs were not found within the same operon. To construct a transporter model, we therefore incorporated the NBD sequence from the structurally characterized maltose ABC importer from *Escherichia coli* (PDB ID: 2R6G (27)) and predicted the SBP–TMD–NBD–ATP complex structure using AlphaFold3 (Fig. 6*A* and S5). The model achieved a predicted template modeling (pTM) score of 0.88, supporting its reliability for structural interpretation. The predicted TMD structure features a large binding pocket with a volume of 1,166 Å^3^ and a depth of 11.9 Å. These dimensions are compatible with the accommodation of CI8 in a conformation similar to that observed in the CIBP-bound state without requiring substantial deformation of the cyclic scaffold (Fig. 6*B*). Moreover, the observation that TdCIBP can bind relatively large linear oligosaccharides such as IG7 in a curved, nearly cyclic conformation suggests a possible structural adaptation toward even linear substrates that facilitates efficient substrate handover to the transporter cavity. Given the comparable binding affinities of TdCIBP for CI7 through CI9, these CIs are likely candidates for transport substrates, as their overall molecular volumes are similar, although the upper limit of oligomerization remains undetermined. Collectively, these findings indicate that *T. decaturensis* harbors an ABC transporter system capable of accommodating and potentially translocating large cyclic and long linear oligosaccharides. Although these observations are consistent with CI and IG transport, direct experimental evidence for substrate translocation and utilization in *T. decaturensis* is currently lacking.

The *TdCIBP* gene is located within a gene cluster that also contains the *CITase* gene (Fig. 1). This cluster includes an additional gene encoding a GH15 46GT, which converts maltodextrin into IGs (16). These findings suggested that *T. decaturensis* produces CIs from maltodextrin through the concerted actions of 46GT and CITase. In addition, *T. decaturensis* harbors genes encoding intracellular dextranase (EC 3.2.1.11) and isomaltose glucohydrolase (EC 3.2.1.205). These glucohydrolases are likely capable of degrading CIs and IGs following their potential uptake *via* the TdCIBP-associated ABC transporter. It is therefore predicted that the maltodextrin metabolic pathway proceeding through CI and IG intermediates provides a selective advantage under carbohydrate-limited conditions, as relatively few bacteria can utilize α-(1→6)-linked glucooligosaccharides. Homologs of *TdCIBP* have also been identified in other CITase-encoding bacteria, including *T. thermocopriae*, *Paenibacillus* sp. 598K, and *P. algidevorans*. These organisms likewise produce CIs from maltodextrin (10, 12, 28, 29), in which CI uptake and intracellular degradation have been experimentally demonstrated (11) to indicate that a conserved maltodextrin metabolic pathway involving CIs as intermediates is broadly maintained in these CI-producing bacteria.

*T. decaturensis* harbors a pseudogene encoding a truncated isomaltodextranase (EC 3.2.1.94) located upstream of the *TdCIBP* gene (Fig. 1). This incomplete isomaltodextranase lacks a portion of the N-terminal catalytic (β/α)_8_-barrel domain and is therefore likely catalytically inactive in dextran degradation. Isomaltodextranase hydrolyzes α-(1→6)-glucans generated by 46GT, producing IG2 from maltodextrin. This activity would be incompatible with the CI-mediated maltodextrin metabolic route proposed for *T. decaturensis*. It is thus plausible that *T. decaturensis* has evolutionarily shifted its α-(1→6)-glucan metabolic intermediate from IG2 to CIs through the acquisition of CITase and CIBP. ABC transporters typically consume two ATP molecules per substrate translocation event; therefore, the uptake of a large carbohydrate, such as CI, is energetically more favorable than the import of multiple small molecules, such as IG2, to acquire an equivalent carbon unit. Therefore, the CI-mediated pathway, coupled with CIBP-dependent transport, likely enables *T. decaturensis* to utilize maltodextrin-derived carbon sources more efficiently than the IG2-based route.

## Experimental procedures

### Preparation of recombinant TdCIBP

The complementary DNA coding TdCIBP was amplified from the genomic DNA of *T. decaurensis* DSM103037 (Leibniz Institute DSMZ–German Collection of Microorganisms and Cell Cultures GmbH) by PCR using the primers (5′-ATGAAAAGGTTTTTTCAATTTATG-3′ and 5′-TTATTTTAAAAATTCATCAATTTC-3′) and KOD FX Neo DNA polymerase (Toyobo). The amplified DNA was used as a template for the second PCR in which the primers (5′-AAGAAGGAGATATACATATGGCCAAGTCGGAAGAAACAAC-3′ and 5′-CAGTGGTGGTGGTGGTGGTGCTCGAGTTTTAAAAATTCATCAATTTC-3′) and Primestar HS DNA polymerase (Takara Bio) were used. The PCR product was inserted into pET - 23a (Novagen) using NEBuilder HiFi DNA Assembly Master Mix (New England Biolabs). The inserted DNA and flanking regions were sequenced using an Applied Biosystems 3130 genetic analyzer (Life Technologies).

Transformant of *E. coli* BL21 (DE3) harboring the expression plasmid for TdCIBP was cultured in 2 l of LB medium containing 100 μg/ml ampicillin at 37 °C until the absorbance reached 0.5 at 600 nm. Recombinant TdCIBP was produced by induction culture with vigorous shaking at 18 °C for 24 h in the presence of 0.1 mM isopropyl β-d-thiogalactopyranoside. Bacterial cells were harvested by centrifugation (9,600*g*, 4 °C, 5 min) and disrupted in 20 mM imidazole–HCl buffer (pH 7.0) containing 0.5 M NaCl by sonication. Cell-free extracts were obtained by centrifugation (13,000*g*, 4 °C, 20 min). Recombinant TdCIBP was purified from the extract by Ni^2+^ affinity column chromatography using a Chelating Sepharose Fast Flow column (Cytiva; 1.5 cm i.d. × 3 cm) equilibrated with 20 mM imidazole–HCl buffer (pH 7.0) containing 0.5 M NaCl. The adsorbed protein was eluted using a linear gradient of 20 to 500 mM imidazole (elution volume, 200 ml) after thoroughly washing the column with equilibrium buffer to elute nonadsorbed proteins. The collected samples were concentrated to approximately 10 ml by ultrafiltration using a Vivaspin YM - 30 concentrator (Sartorius) and subjected to gel filtration column chromatography using a Toyopearl HW - 55S column (Tosoh; 2.6 cm i.d. × 100 cm) equilibrated with 10 mM Hepes–NaOH buffer (pH 7.0). The concentration of purified TdCIBP was determined by amino acid analysis using an amino acid analyzer L-8900 (Hitachi High-Technologies) after complete hydrolysis of the purified protein in 6 M HCl at 110 °C for 24 h.

### SEC–MALS

SEC–MALS was measured using the Alliance 2695 HPLC system (Waters) coupled to a Dawn Heleos II MALS detector (Wyatt Technology) and a 2414 refractive detector (Waters). KW - 803 protein (Shodex) was separated before the MALS analysis at a flow rate of 0.5 ml/min. The running buffer contained 10 mM Hepes–NaOH buffer (pH 7.2) and 150 mM NaCl. The absolute molecular mass was calculated by analyzing the scattering data using the ASTRA analysis software package (Wyatt Technology).

### ITC measurement

The thermodynamic parameters for the binding between TdCIBP and each ligand sugar were determined using ITC with a MicroCal PEAQ-ITC Automated Instrument (Malvern Panalytical). All protein samples were dialyzed against 10 mM Hepes–NaOH buffer (pH 7.0) at 4 °C for 18 h. Titrants (CI9, CI8, CI7, IG7, IG6, IG5, IG4, IG3, IG2, d-glucose, and γ-CD) were dissolved in the dialysis buffer used. The cell and syringe were filled with 15 μm TdCIBP and 150 μm titrants (CI9, CI8, CI7, IG7, IG6, IG5, IG4, IG3, IG2, d-glucose, and γ-CD). Each experiment consisted of a single 0.4-μl injection over 0.8 s of the titrant into the SBP solution at 25 °C. The spacing time was 150 s, the reference power was 5 μcal/s, and the stirring speed was set to 750 rpm. For each experiment, the data from the ITC buffer injection into the receptor in the cell were used for blank subtraction. Data analysis was performed using MicroCal PEAQ-ITC software, version 1.41 (Malvern Panalytical) with a one-set-sites model. The following sugars were used as ligands: CI7 and CI9, kindly gifted from emeritus professor Kazumi Funane (Yamanashi University); CI8, kindly gifted from past professor Tetsuya Oguma (Niigata Agro-Food University); IG2 and IG3 (Tokyo Chemical Industry); IG4–IG7 (Seikagaku); and d-glucose and γ-CD (Nacalai Tesque).

### Crystallization, data collection, data processing, phasing, and refinement

Crystals of TdCIBP in complex with CI8 and IG7 were prepared by cocrystallization using the sitting-drop vapor-diffusion method. Appropriate crystals of the TdCIBP–CI8 complex were obtained within one month at 20 °C with a drop (1.5 μl) containing 9.2 mg/ml TdCIBP, 2 mM CI8, 0.1 M NaCl, 50 mM phosphate citrate (pH 4.2), and 10% (w/v) polyethylene glycol 8000 (Sigma). Appropriate crystals of the TdCIBP–IG7 complex were obtained within 2 weeks at 20 °C with a drop (1.5 μl) containing 9.2 mg/ml TdCIBP, 20 mM IG7, 0.2 M Li_2_SO_4_, 0.1 M sodium acetate (pH 4.5), and 30% (w/v) polyethylene glycol 8000.

### Data collection, data processing, phasing, and refinement

X-ray diffraction data for TdCIBP were automatically collected by the ZOO system at beamline BL45XU of SPring - 8 (30, 31). The X-ray wavelength was set at 1.0000 Å. The diffraction images were processed using XDS (32). The TdCIBP structure was solved by molecular replacement using PHENIX Phaser (33) with the predicted model from ColabFold (34) as a search model. During the refinement, the N-terminal residues (26–52 for the CI8 complex and 26–44 for the IG7 complex) were removed because of a poor electron density map. The structure was refined using PHENIX (33) and Coot (35). Data collection and refinement statistics are summarized in Table 2. A composite omit map was calculated using PHENIX. Molecular graphic images were prepared using PyMOL (Schrodinger LLC).

### Prediction of the TdCIBP–ABC transporter complex and cavity identification

The three-dimensional structure of the TdCIBP–ABC transporter complex, including the TdCIBP, TMDs, NBDs, and ATP, was predicted using the ProteinX server (36) (https://protenix-server.com; DOI: https://doi.org/10.1101/2025.01.08.631967). The NBD sequence used for the prediction was derived from the crystal structure of the maltose transporter (PDB ID: 2R6G). The amino acid sequences of each component were submitted to the server, and the prediction was conducted using default parameters. The confidence of the predicted model was evaluated using the pTM and interface pTM scores, which were 0.88 and 0.87, respectively. The substrate-binding cavity was identified using the POCASA web server (37) (https://g6altair.sci.hokudai.ac.jp/g6/service/pocasa/) with the default parameters, and the detected pocket was further inspected by superimposing CI8 and IG7 into the predicted cavity.

### Comparison of the CI8 structure using the SAXS data

For comparison with the solution structure of free CI8, previously reported SAXS scattering data were used. The scattering curve of free CI8 was obtained from the published dataset reported (20) and replotted using RAW (38) without reprocessing of the original experimental data. For CI8 bound to TdCIBP, theoretical SAXS scattering curves were calculated from the atomic coordinates of the crystal structure using CRYSOL (39) and plotted using RAW. The radius of gyration (Guinier *R*g) for the TdCIBP-bound CI8 was calculated from the atomic coordinates (39).

## Data availability

The atomic coordinates have been deposited in the PDB. Deposition IDs are 9XF5 for TdCIBP/CI8 and 9XFU for TdCIBP/IG7.

## Supporting information

This article contains supporting information.

## Conflict of interest

The authors declare that they have no conflicts of interest with the contents of this article.
