# Supplementary material for: Insights into the recognition of cyclic α-(1→6)-glucan by a solute-binding protein of an ABC transporter from Tepidibacillus decaturensis
Source: J Biol Chem. 2026 Mar 4;302(5):111346. doi: 10.1016/j.jbc.2026.111346 (PMC13084667; doi:10.1016/j.jbc.2026.111346)
Supplement: Supporting Information [file mmc1.pdf]

SUPPORTING INFORMATION

Insights into the recognition of cyclic α -(1→6)-glucan by a solute-binding protein of an ABC transporter from *Tepidibacillus decaturensis*

Shiho Takei[1], Wataru Saburi[2], Min Yao[1], Haruhide Mori [2], Toyoyuki Ose[1]

1 Faculty of Advanced Life Science, Hokkaido University, Sapporo, 060-0810 Japan

2 Research Faculty of Agriculture, Hokkaido University, Sapporo, 060-8589 Japan

Table of Contents

Figure S1 SEC-MALS result with recombinant TdCIBP.................................. S2

Figure S2 ITC measurements of TdCIBP binding to various oligosaccharides.. S3

Figure S3 Structural comparison of CI8.. S4

Figure S4 Details of CI8 and IG7 structure.. S5

Figure S5 AlphaFold3 Predicted structure of SBP, TMDs, NBDs and ATP...... S6

Table S1 Interactions in TdCIBP between CI8/IG7... S7

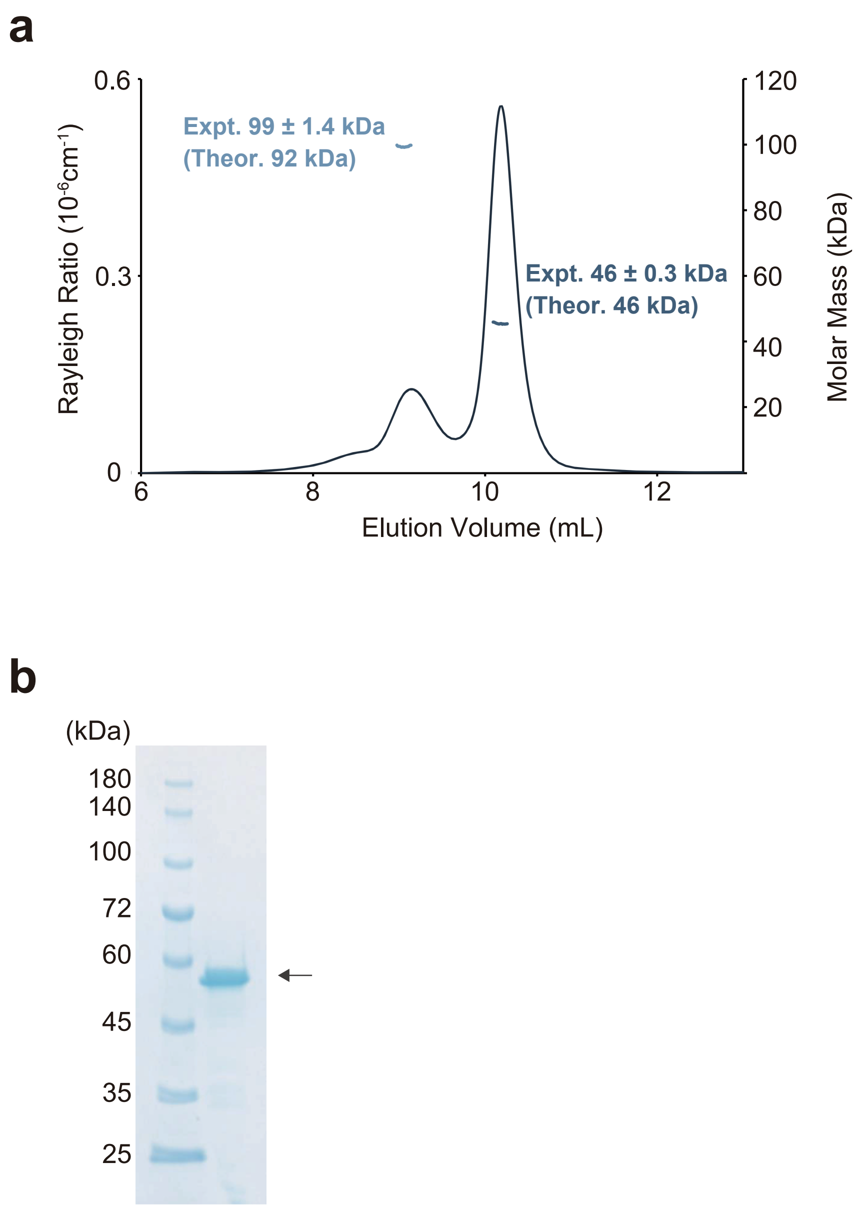

Figure S1 SEC-MALS result with recombinant TdCIBP. (a) SEC–MALS analysis of TdCIBP. Measured molecular masses are shown, and theoretical masses (calculated from amino acid sequences) are indicated with brackets. The values in each frame, ± error, are the estimated molecular masses at peak position analyzed using the ASTRA analysis software package (Wyatt Technology). (b) SDS-PAGE analysis of TdCIBP.

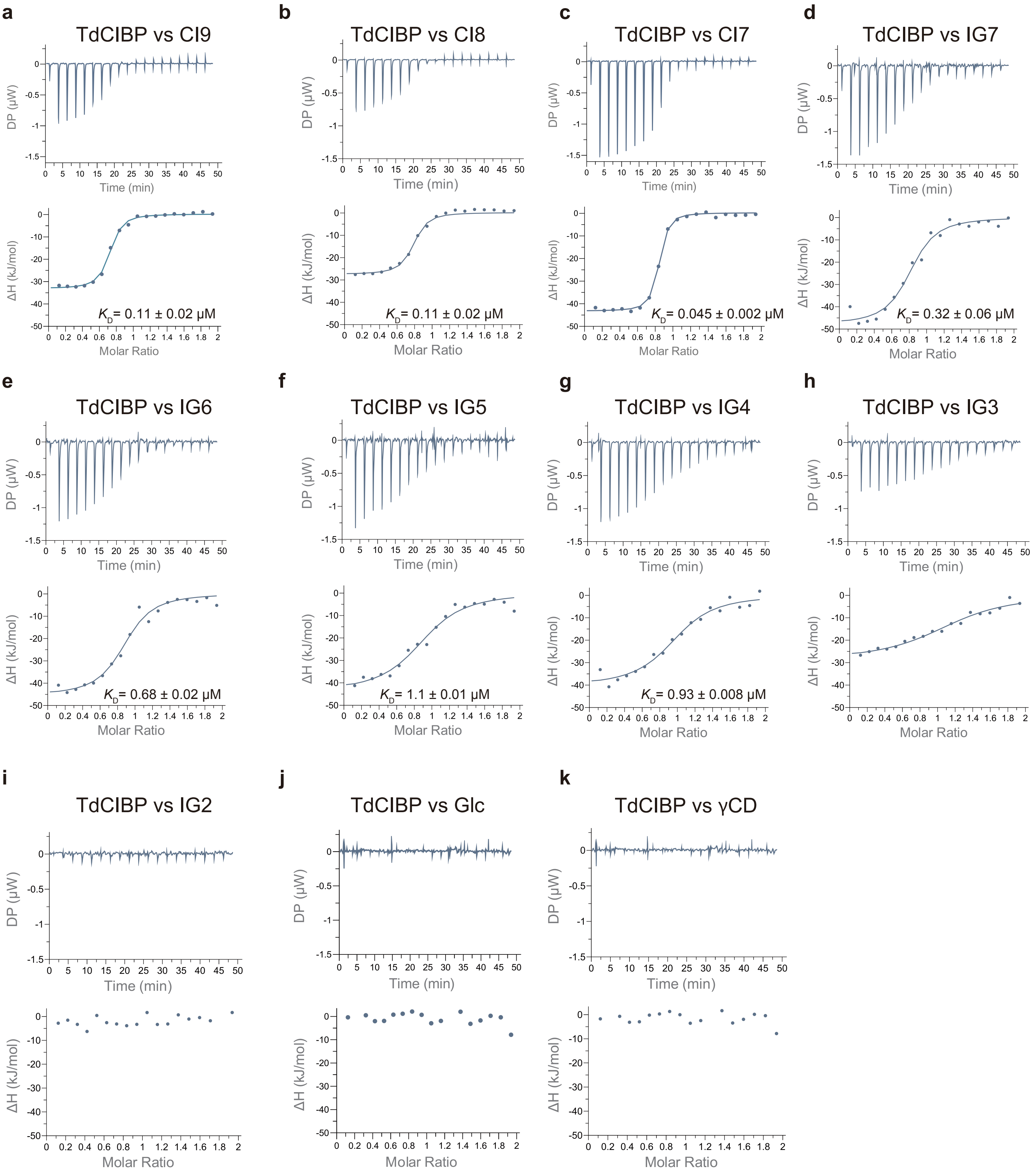

Figure S2. ITC measurements of TdCIBP binding to various oligosaccharides. The upper panels show the representative titration thermograms after blank value subtraction, and the lower panels show the data integration with fitted curves (one set of sites model). TdCIBP was titrated with (a) CI9, (b) CI8, (c) CI7, (d) IG7, (e) IG6, (f) IG5, (g) IG4, (h) IG3, (i) IG2, (j) D-glucose, and (k) γ-CD.

a

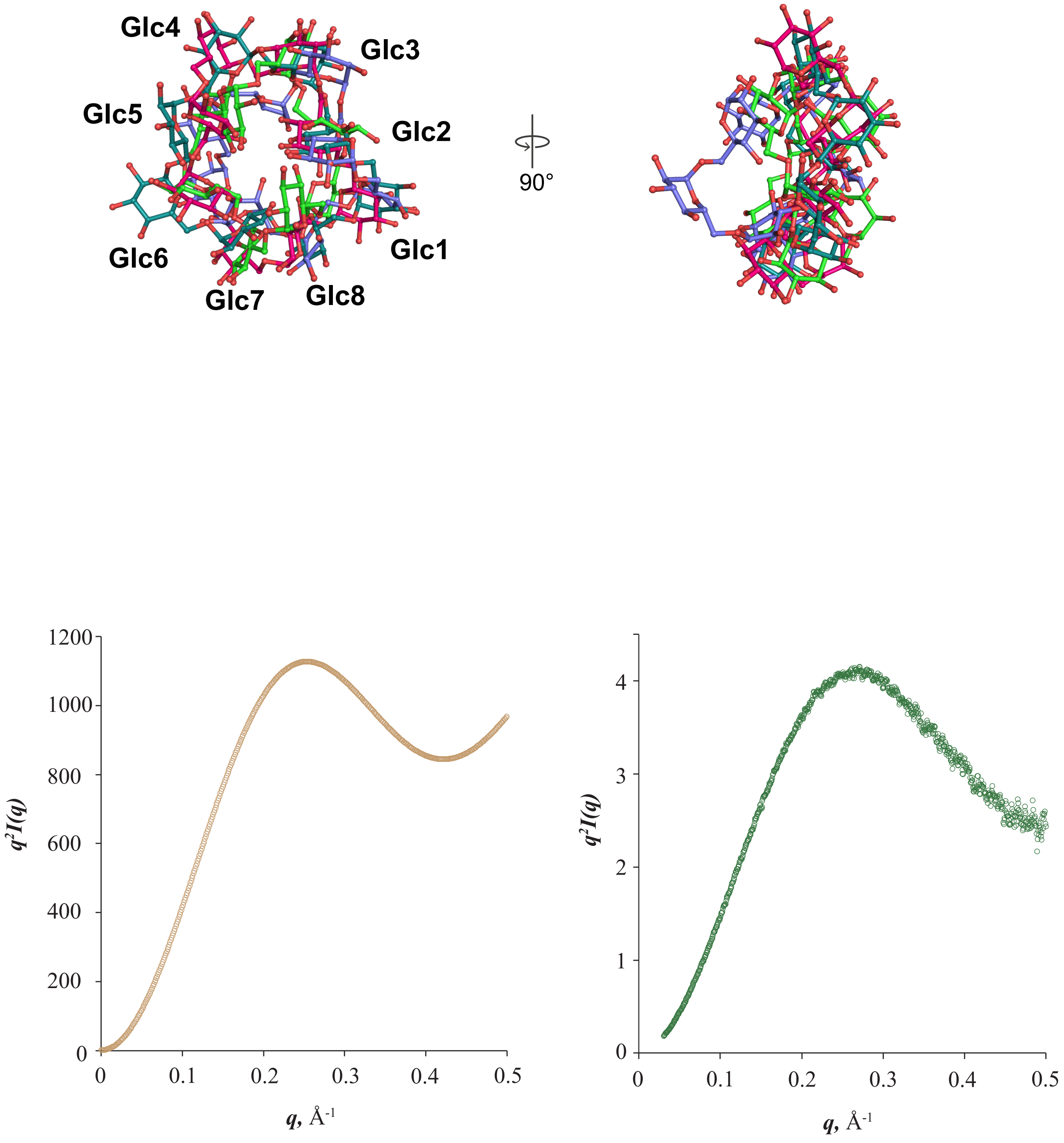

Figure S3 Structural comparison of CI8. (a) Superimposition of CI8 structures derived from X-ray crystallography. CI8 bound to CIBP is shown in cyan, while CI8 molecules from PDB ID: 3WNO are shown in pink (chain C), green (chain D), and purple (chain E).
(b) The left panel shows Kratky plot generated from calculated scattering curves computed using CRYSOL[46] and RAW [47] based on the X-ray crystallographic structures of CI8 bound to CIBP. The right panel shows Kratky plot of SAXS data for CI8.

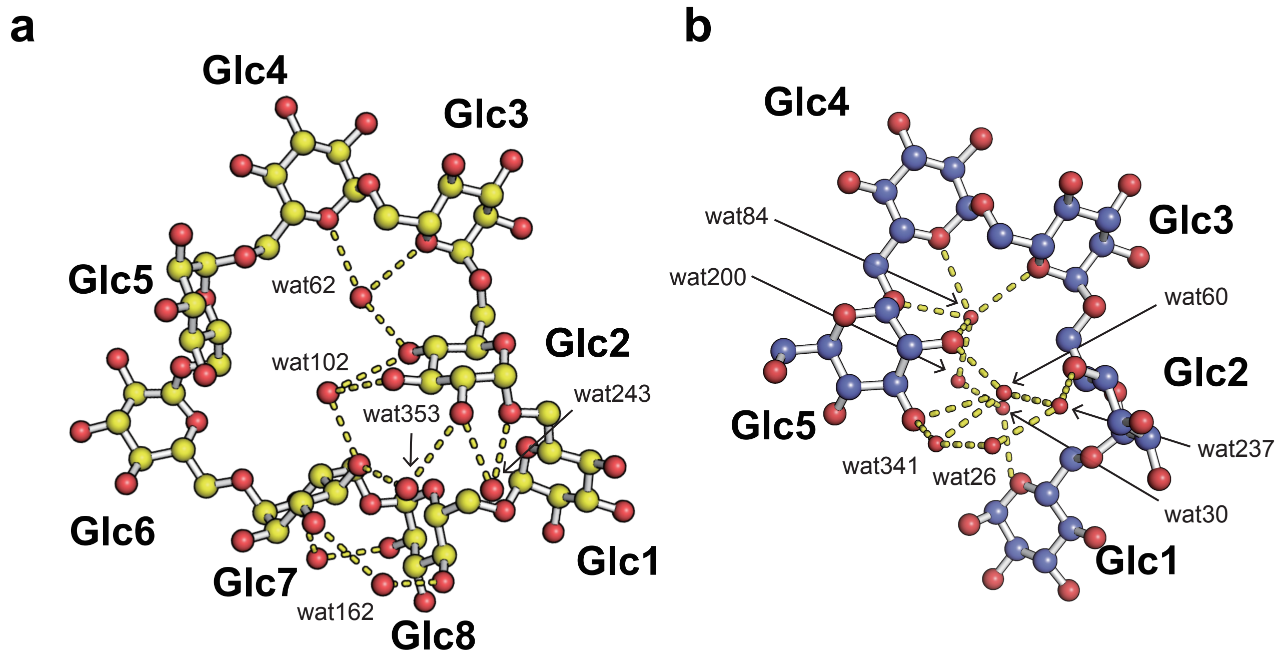

Figure S4 Intramolecular hydrogen bonds within CI8 (a) and IG7 (b). CI8 and G7 are shown using ball-and-stick representation, with intramolecular hydrogen bonds indicated by dashed lines. Hydrogen bonds are drawn using a threshold of 3.3 Å.

a

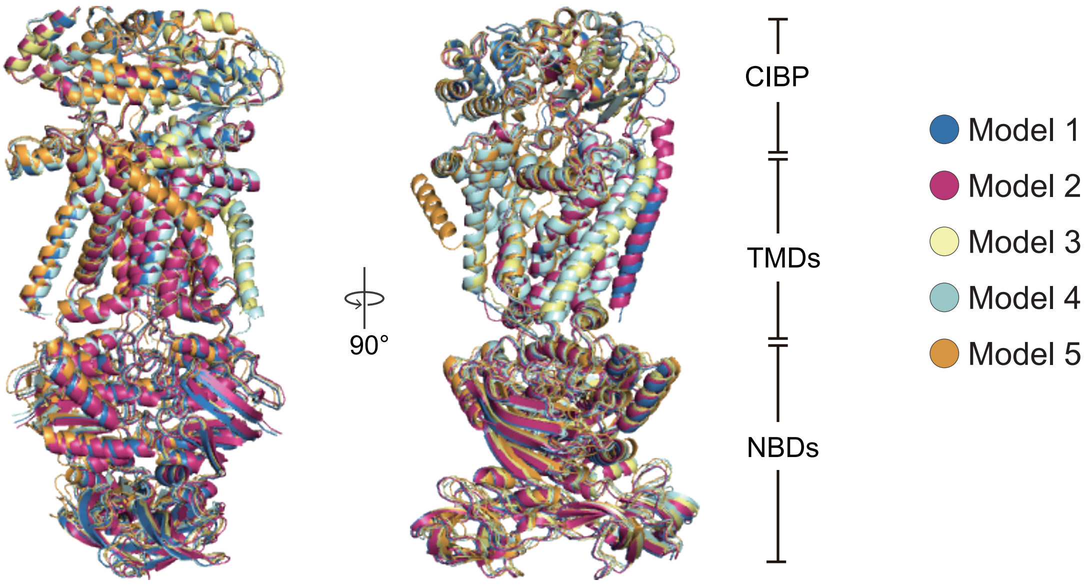

b

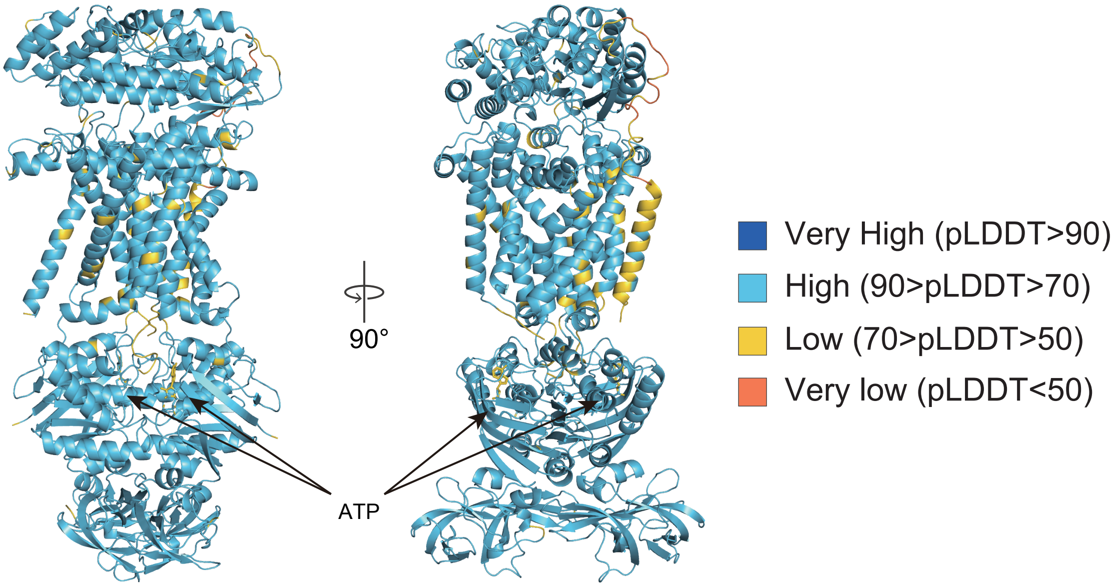

Figure S5 AlphaFold3 predicted structure of the CIBP/TMDs/NBDs/ATP complex from *T. decaturensis* and *E. coli.* **(a)** Superposition of the five AlphaFold3-predicted models, shown in two orthogonal views (90° rotation). **(b)** Representative model colored by pLDDT score. ATP molecules are shown. Two orthogonal views (90° rotation) are displayed.

Possible hydrogen bond (<3.3 A)							
	Glc	Atom	Target	Atom	Distance(Å)		
CI8	1	O4	Asp64	OD1	2.8		
	3	O2	Glu66	OE1	2.7		
	3	O2	Glu66	OE2	3.1		
	3	O2	Ser63	OG	2.7		
	3	O3	Arg115	NH2	3		
	3	O4	Glu322	OE2	2.7		
	3	O4	Thr61	OG1	2.8		
	3	O4	Arg115	NH1	3.1		
	4	O2	Asn168	ND2	2.9		
	4	O3	Asp117	OD1	2.7		
	4	O4	Arg389	NH1	2.8		
	4	O4	Arg389	NH2	3.2		
	5	O2	Tyr94	OH	2.9		
	8	O2	Trp275	N	3.2		
Possible water-mediated hydrogen bond(<3.3 A)							
	Glc	Atom	water	Target	Atom	Distance(Å)	Distance(Å)
CI8	2	O5	water40	Thr61	O2	2.8	2.7
	3	O4	water43	Glu322	OE2	3.3	2.8
	6	O3	water68	Asp396	OD1	2.7	3
Possible sugar–water hydrogen bond(<3.3 A)							
	Glc	Atom	water	Target sugar	atom		
CI8	1	O6	water243	Glc2	O2	2.8	3.2
	2	O4	water62	Glc3	O5	2.9	2.7
	2	O4	water62	Glc4	O5	2.9	3.2
	2	O3	water102	Glc7	O4	2.9	2.7
	2	O2	water353	Glc7	O4	3.3	2.7
	3	O5	water62	Glc4	O5	2.7	3.2
	8	O5	water60	Glc2	O4	3	2.9
	7	O5	water61	Glc8	O2	2.8	2.9
	7	O3	water162	Glc8	O4	3.3	2.7

C-C contacts (<4.5 A)					
	Glc	Atom	Target	Atom	Distance(Å)
CI8	1	C1	Trp275	CH2	4.2
	1	C1		CZ3	3.8
	1	C1		CE3	4.2
	3	C1	Trp290	CG	3.7
	3	C1		CD2	3.4
	3	C1		CD1	4
	3	C1		CE2	3.6
	3	C1		CE3	3.8
	3	C1		CZ3	4.2
	3	C1		CH2	4.3
	3	C1		CZ2	4.1
	3	C2		CG	4
	3	C2		CD2	3.6
	3	C2		CE2	4.2
	3	C2		CE3	3.4
	3	C2		CZ3	4
	3	C2		CH2	4.5
	4	C2	Trp217	CH2	4.1
	4	C2		CZ2	4.4
	4	C3		CH2	4.5
	4	C4		CD2	4.2
	4	C4		CE2	4.3
	4	C4		CE3	4
	4	C4		CZ3	4
	4	C4		CH2	4.1
	4	C4		CZ2	4.3
	4	C5		CE2	4.4
	5	C1	Trp217	CG	3.9
	5	C1		CD1	4.1
	5	C1		CD2	4.3
	5	C5		CG	4.5
	5	C5		CD1	4.1
	8	C1	Trp275	CG	4
	8	C1		CD1	4
	8	C1		CD2	4
	8	C1		CE2	4.1
	8	C2		CG	3.7
	8	C2		CD1	4
	8	C2		CD2	4.1

Possible hydrogen bond (<3.3 A)							
	Glc	Atom	Target	atom	Distance(Å)		
IG7	2	O3	Arg91	NH2	3.1		
	3	O2	Glu66	OE2	2.6		
	3	O2	Ser63	OG	2.8		
	3	O3	Glu66	OE1	2.7		
	3	O3	Arg115	NH2	3		
	3	O4	Thr61	OG1	2.8		
	3	O4	Glu322	OE2	2.8		
	4	O2	Asn168	ND2	2.9		
	4	O2	Glu322	OE2	2.9		
	4	O3	Asp117	OD1	2.7		
	4	O4	Asp117	OD2	2.6		
	4	O4	Arg389	NH1	2.8		
Possible water-mediated hydrogen bond(<3.3 A)							
	Glc	Atom	water	Target	atom	Distance(Å)	Distance(Å)
IG7	2	O2	water767	Asp64	OD1	2.6	3
	2	O3	water767	Asp64	OD2	2.6	2.7
	2	O4	water248	Thr61	O	2.6	2.7
	3	O3	water25	Asn168	ND2	3	2.8
	3	O3	water25	Glu322	OE1	3	2.7
	5	O5	water111	Tyr94	OH	3	3
Possible sugar–water hydrogen bond(<3.3 A)							
	Glc	Atom	water	Target	atom	Distance(Å)	Distance(Å)
IG7	2	O4	water60	GLC5	O2	2.5	2.7
	3	O5	water84	Glc4	O5	2.9	2.8
	3	O5	water84	Glc4	O6	2.9	3
	3	O5	water84	Glc5	O2	2.9	2.9

C-C contacts (<4.5 A)					
	Glc	Atom	Target	Atom	Distance(Å)
IG7	1	C1	Trp275	CE2	4.5
	1	C2		CG	3.6
	1	C2		CD1	3.8
	1	C2		CD2	3.9
	1	C2		CE2	4.2
	1	C3		CG	4.3
	1	C3		CD2	4.3
	1	C3		CE3	4.5
	1	C4		CG	4.5
	1	C4		CD2	4
	1	C4		CE2	4.3
	1	C4		CE3	3.9
	1	C4		CZ3	4.2
	1	C4		CH2	4.5
	1	C5		CD2	4.5
	1	C5		CZ2	4.4
	1	C5		CE2	4.4
	1	C6		CZ3	4.2
	1	C6		CH2	3.8
	1	C6		CZ2	3.8
	1	C6		CE2	4.2
	3	C1	Trp290	CG	3.7
	3	C1		CD1	4
	3	C1		CZ2	4
	3	C1		CD2	3.4
	3	C1		CE3	3.8
	3	C1		CZ3	4.2
	3	C1		CH2	4.3
	3	C1		CZ2	4
	3	C1		CE2	3.5
	3	C2		CG	4
	3	C2		CD2	3.6
	3	C2		CE2	4.2
	3	C2		CE3	3.4
	3	C2		CZ3	3.9
	3	C2		CH2	4.5
	4	C1	Trp290	CH2	4.4
	4	C2	Trp217	CZ2	4.4
	4	C2	Trp217	CH2	4.1
	5	C1	Tyr94	CE1	3.7
	5	C1	Tyr94	CZ	3.9
	5	C1	Tyr94	CE2	4.3
	5	C1	Tyr94	CD1	4

Table S1 Interactions in TdCIBP between CI8/IG7
